# Cell-permeable iron inhibits vascular endothelial growth factor receptor-2 signaling and tumor angiogenesis

**DOI:** 10.18632/oncotarget.11689

**Published:** 2016-08-30

**Authors:** Devika Kir, Manju Saluja, Shrey Modi, Annapoorna Venkatachalam, Erica Schnettler, Sabita Roy, Sundaram Ramakrishnan

**Affiliations:** ^1^ Department of Pharmacology, Masonic Comprehensive Cancer Center, Minneapolis, MN 55455, USA; ^2^ Department of Surgery, University of Minnesota, Minneapolis, MN 55455, USA; ^3^ Present address: Department of Surgery, University of Miami, FL 33136, USA

**Keywords:** cell-permeable iron, ferric ammonium citrate, angiogenesis, vascular endothelial growth factor, receptor phosphorylation

## Abstract

Angiogenesis is important for tumor growth and metastasis. Hypoxia in tumors drives this angiogenic response by stabilizing Hypoxia Inducible Factors (HIF) and target genes like Vascular Endothelial Growth Factor (VEGF). HIF stability is regulated by Prolylhydroxylases (PHD)-mediated modification. Iron is an important cofactor in regulating the enzymatic activity of PHDs. Reducing intracellular iron, for instance, mimics hypoxia and induces a pro-angiogenic response. It is hypothesized that increasing the intracellular iron levels will have an opposite, anti-angiogenic effect. We tested this hypothesis by perturbing iron homeostasis in endothelial cells using a unique form of iron, Ferric Ammonium Citrate (FAC). FAC is a cell-permeable form of iron, which can passively enter into cells bypassing the transferrin receptor mediated uptake of transferrin-bound iron. Our studies show that FAC does not decrease the levels of HIF-1α and HIF-2α in endothelial cells but inhibits the autocrine stimulation of VEGF-Vascular Endothelial Growth Factor Receptor-2 (VEGFR-2) system by blocking receptor tyrosine kinase phosphorylation. FAC inhibits VEGF-induced endothelial cell proliferation, migration, tube formation and sprouting. Finally, systemic administration of FAC inhibits VEGF and tumor cell-induced angiogenesis *in vivo*. In conclusion, our studies show that cell-permeable iron attenuates VEGFR-2 mediated signaling and inhibits tumor angiogenesis.

## INTRODUCTION

Angiogenesis, the formation of new blood vessels from pre-existing vasculature, is a pathophysiological process necessary for tumor growth and metastases. [1]. Reduced oxygen availability (hypoxia) is the major stimulus for angiogenesis. Hypoxia increases the cellular levels of HIF (HIF-1, HIF-2 and HIF-3) family of transcription factors. HIFs transcriptionally activate target genes such as VEGF, which then drive angiogenesis [2–4]. Under normal oxygen levels, HIF-1α and HIF-2α are maintained at very low levels by proteasomal degradation after PHD-mediated modification. PHDs are integral to HIF regulation and function as the oxygen sensing molecular switches. Iron is one of the cofactors in regulating the enzymatic activity of PHDs. This is reflected by the stabilization of HIF-1α and HIF-2α in the presence of an iron chelator, Deferoxamine (DFX) [5–7]. Molecular iron is also essential for various cellular functions and metabolic processes. While trace amounts of iron is required to maintain cellular homeostasis, excessive iron is toxic to cells by the generation of reactive hydroxyl and oxygen radicals by Fenton/Haber Weiss reaction [8]. Iron levels are therefore tightly regulated in cells by controlling its uptake, storage and export. Iron is transported into cells by receptor-mediated endocytosis of transferrin [9]. We have previously shown that Dynamin-2 (a GTPase that mediates endocytosis) is down regulated during hypoxia, which lowers the intracellular iron levels in cells with concomitant stabilization of HIF-1α and HIF-2α [6]. FAC is a cell-permeable form iron which can passively enter into cells bypassing the transferrin receptor mediated uptake of transferrin-bound iron [10]. In tumor cells, FAC was able to counteract the effect of Dynamin-2 inhibition on HIF-1α [6]. Therefore, we investigated the effect of FAC in perturbing iron homeostasis in endothelial cells and its effect on angiogenesis. While the tumor cells secrete VEGF as a paracrine growth factor under hypoxia, endothelial cells are activated by VEGF-mediated autocrine stimulation. Interestingly, FAC treatment did not decrease the levels of HIF-1α and HIF-2α in endothelial cells but inhibited the autocrine stimulation of VEGF-VEGFR-2 system by blocking receptor tyrosine kinase phosphorylation. Further, our studies show that systemic treatment of mice with FAC inhibits VEGF and tumor cell induced-angiogenesis *in vivo*.

## RESULTS

### Cell-permeable iron inhibits VEGF-A induced proliferation of endothelial cells

**Figure 1:**
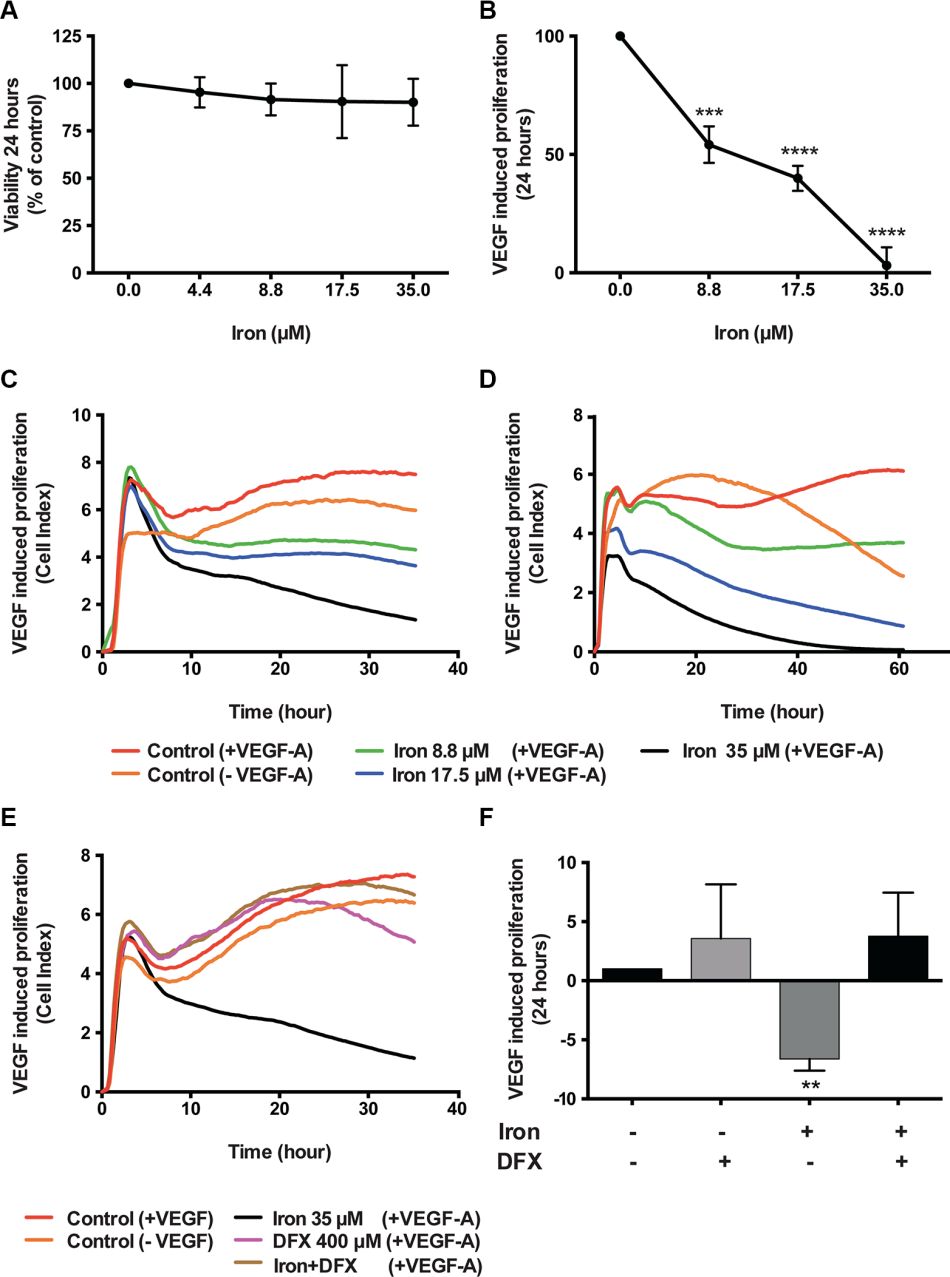
Cell-permeable iron inhibits VEGF-A induced endothelial cell proliferation Viability of endothelial cells was determined by CCK-8 assay kit. (**A**) Data show the effect of cell-permeable iron on quiescent HUVEC-I. (**B**) Inhibition of VEGF-A induced proliferation (viability) of growth factor starved HUVEC-I by cell-permeable iron. The cells were stimulated to proliferate by VEGF-A (100 ng/ml) for 24 hours. All experiments were performed in triplicates and data represent mean ± SD from three independent experiments. ***P < 0.001, ****P < 0.0001. Representative real-time tracings of impedance measurements (VEGF-A induced proliferation) in the presence and absence of cell-permeable iron are shown. (**C**) HUVEC-I and (**D**) MVEC. (**E**) Representative real-time tracing of proliferation in HUVEC-I induced by VEGF-A (100 ng/ml) in the presence of cell-permeable iron, DFX or both is shown. (**F**) The histogram represents VEGF-A induced proliferation in HUVEC-I in the presence of cell-permeable iron, DFX or both. Data represent mean ± SD of two independent real-time experiments. **P < 0.01.

Effect of cell-permeable iron on endothelial cell proliferation was determined using Human Umbilical Vein Endothelial Cells immortalized with SV40 antigen (HUVEC-I) and microvascular endothelial cells (MVEC). Initial studies showed a differential sensitivity of HUVEC-I to cell-permeable iron depending on proliferation status. Quiescent endothelial cell viability was not affected by FAC even at a concentration of 35 μM (Figure 1A). However, when the quiescent cells were stimulated to proliferate with VEGF-A, cell-permeable iron treatment showed a concentration dependent inhibition of endothelial cell proliferation as determined by viability (Figure 1B). VEGF-induced proliferation was inhibited by 50% at 9 μM and complete inhibition was observed at 35 μM. Then we determined the effect of cell permeable iron on VEGF-induced proliferation of HUVEC-I and MVEC in xCELLigence system, which measures cell attachment and growth by electrical impedance measurements in real-time. These studies showed that both HUVEC-I and MVEC were inhibited by cell permeable iron. Within ten hours, endothelial cells exposed to iron showed a concentration-dependent inhibition in electrical impedance (Cell index). Representative real-time tracings are shown in Figure 1C and Figure 1D. These results were confirmed in two independent experiments.

Next, we wanted to test whether iron is the active component of FAC responsible for the inhibition of VEGF-A induced endothelial proliferation. For this purpose, an iron chelator, DFX was used. VEGF-A induced endothelial (HUVEC-I) proliferation in the presence or absence of DFX was monitored in real-time. Representative tracings (Figure 1E) show that chelation of iron by DFX reversed FAC mediated inhibition of endothelial cell proliferation. Figure 1F shows the effect of FAC, DFX or a combination of both on VEGF-A induced endothelial proliferation. Data represent mean of cell indices from six-eight different wells from two independent experiments. These results confirm that cell permeable iron, FAC, inhibits VEGF-induced endothelial cell proliferation.

**Figure 2:**
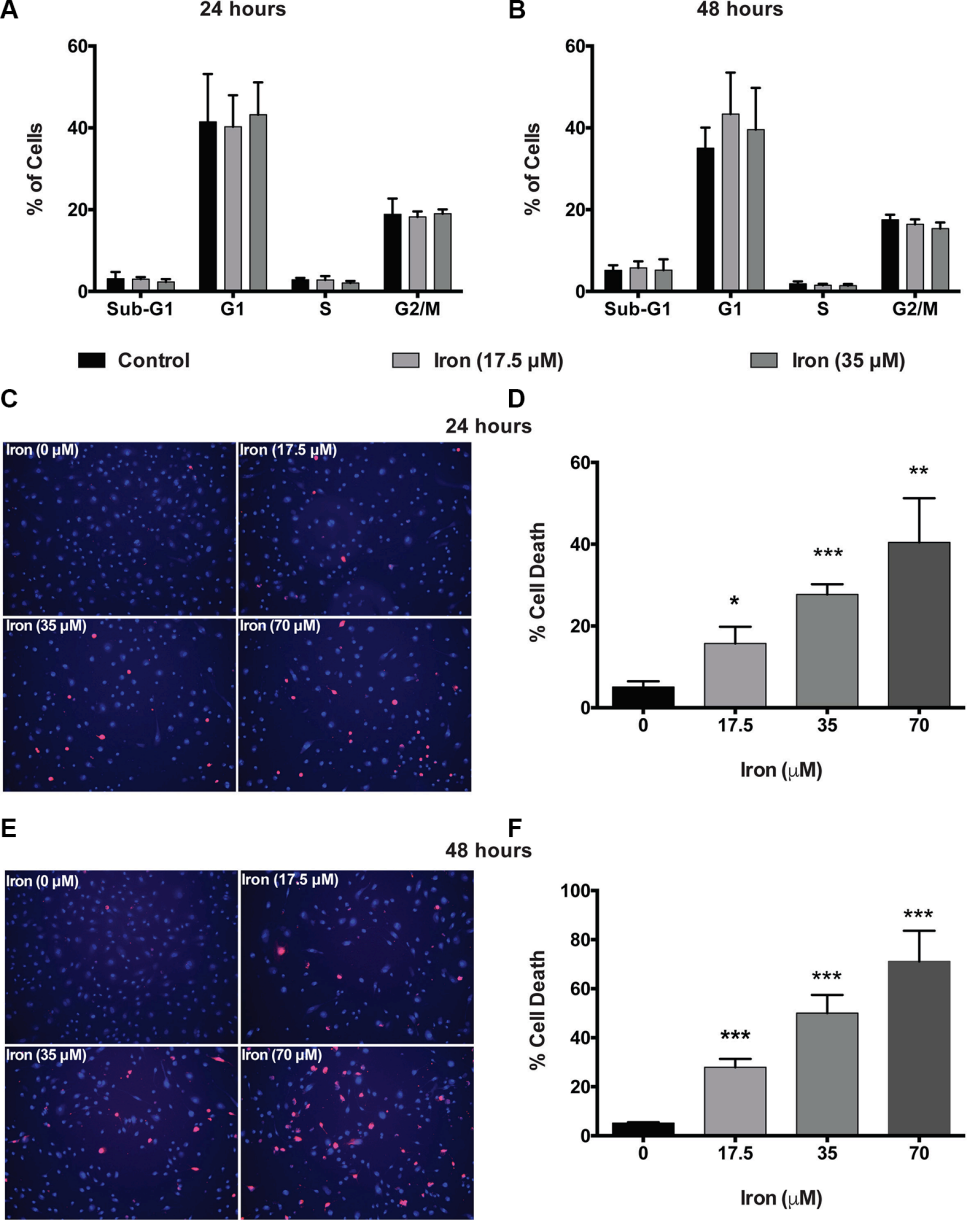
Cell-permeable iron induces cell death in VEGF-A stimulated endothelial cells Cell-cycle analyses of HUVEC-I treated with cell-permeable iron (17.5 μM and 35 μM) were carried out by flow cytometry. (**A**) Cells treated for 24 hours. (**B**) Cells treated for 48 hours. Data represent mean ± SD of four independent experiments. (**C**) Representative images of PI stained HUVEC-I stimulated with VEGF-A (100 ng/ml) in the presence of cell-permeable iron for 24 hours. Hoescht-33342 (blue, total cells) and PI (red)-stained (dead cells) are shown (10× magnification). (**D**) The histogram represents cell death analyses from three independent experiments. Cell death was calculated as a percentage of PI positive nuclei from the total number of Hoescht-33342 positive nuclei (blue) per field. Data represent mean ± SD. *P < 0.05, **P < 0.01, ***P < 0.001. (**E**) Representative images of HUVEC-I stimulated with VEGF-A (100 ng/ml) in the presence of cell-permeable iron for 48 hours. Dead cells were identified by PI staining. (**F**) The histogram represents cumulative data of cell death analyses from three independent experiments. Data represent mean ± SD. ***P < 0.001.

**Figure 3:**
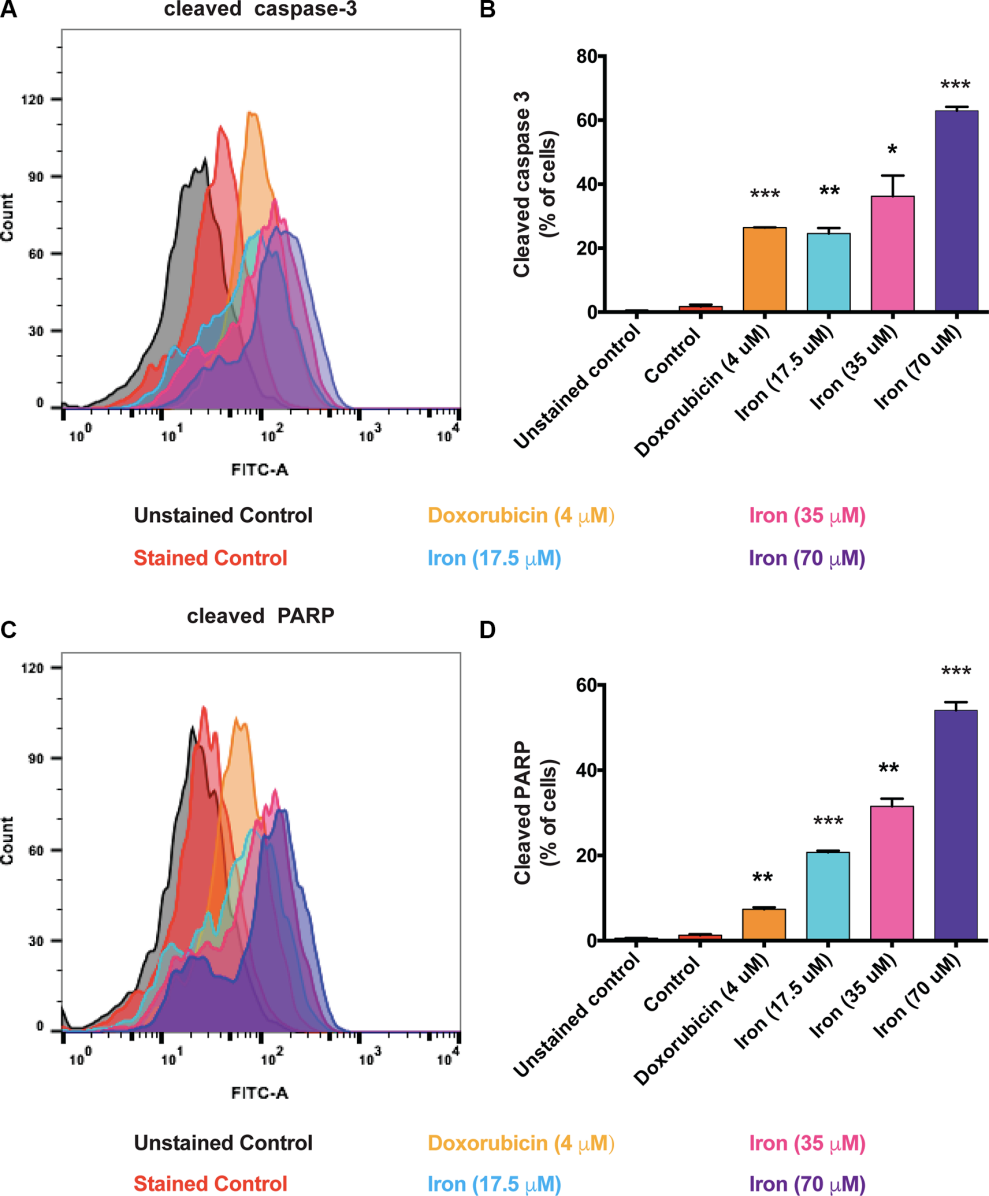
Cell-permeable iron induces apoptosis in VEGF-A stimulated endothelial cells Flow cytometric analysis was used to detect the levels of cleaved caspase-3 and cleaved PARP in the presence of cell-permeable iron (35 μM) for 48 hours in VEGF-A (100 ng/ml) stimulated HUVEC-I. (**A**) Representative histogram of cells positive for cleaved caspase-3 is shown. (**B**) Cumulative data of cleaved caspase-3-positive cells from two independent experiments are shown. (**C**) Representative flow cytometric analysis of cells positive for cleaved PARP positive cells. (**D**) Cumulative data represent mean ± SD from two independent experiments. *P < 0.05, **P < 0.01, ***P < 0.001.

In order to investigate the mechanism of inhibition of VEGF-A induced cellular proliferation, we evaluated the effect of cell-permeable iron on cell cycle (HUVEC-I). Cell-permeable iron treatment did not inhibit progression of cell cycle in endothelial cells (HUVEC-I) (Figure 2A and 2B). However, iron treatment induced cell death in VEGF-A stimulated endothelial cells (HUVEC-I) as shown by loss of membrane integrity and Propidium Iodide (PI) uptake in a concentration and time dependent manner (Figure 2C–2F). Hence, cell-permeable iron inhibits VEGF-induced proliferation in endothelial cells by inducing cell death. In order to ascertain the mechanism of cell death, markers of apoptosis such as cleaved caspase-3 and cleaved Poly ADP-Ribose Polymerase (PARP), were determined by flow cytometric analysis. Doxorubicin (4 μM) was used as a positive control. Cell-permeable iron, after 48 hours of treatment, significantly increased the number of cells positive for cleaved caspase-3 (Figure 3A and 3B) and cleaved PARP (Figure 3C and 3D) in VEGF-A stimulated endothelial cells (HUVEC-I) in a concentration dependent manner. More than 30% of cells were positive for cleaved caspase-3 and PARP when treated with 35 μM iron. These studies confirm that cell permeable iron induces apoptosis of endothelial cells when stimulated with VEGF.

### Cell-permeable iron inhibits VEGF-A induced migration and tube formation of endothelial cells

**Figure 4:**
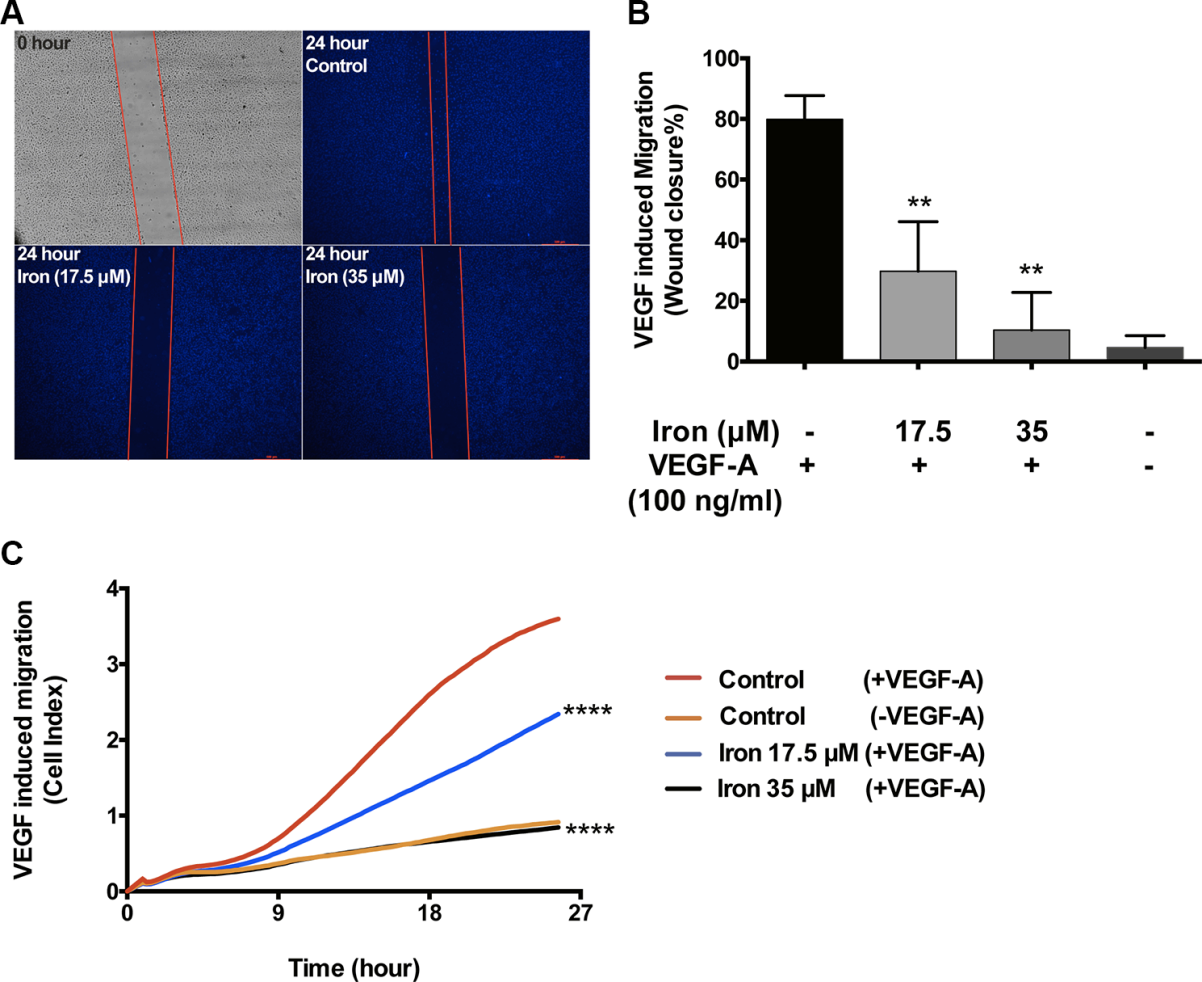
Cell-permeable iron inhibits VEGF-A induced endothelial migration (**A**) Scratch wound assays were carried out to determine the effect of iron on VEGF-A (100 ng/ml) induced migration in HUVEC-I. Representative zero hour (immediately after the scratch) and twenty-four hour images of the scratch in the presence of iron (17.5 μM, 35 μM) are shown (2.5× magnification). (**B**) The histogram represents migration as percent closure of the wound area 24-hours after scratch wound. Data represent mean ± SD of three independent experiments. **P < 0.01. (**C**) Represents real-time migration of HUVEC-I towards a gradient of VEGF-A (100 ng/ml) in the presence of cell-permeable iron (17.5 μM, 35 μM). Electrical impedance (Cell index) was determined in real-time using CIM plates in an xCELLigence system. Data points are a mean of quadruplicate cultures. Statistical significance was determined from mean ± SD. Significant inhibition was seen as early as nine hours after treatment with cell-permeable iron. ****P < 0.0001.

After establishing the effect of cell-permeable iron on VEGF-A induced endothelial proliferation, we carried out experiments to study the effect of iron on endothelial migration. Endothelial cells (HUVEC-I) were stimulated to migrate by VEGF-A in scratch wound assays. The control endothelial cells efficiently closed the wound by eighty percent after 24 hours in culture. In the presence of cell-permeable iron (17.5 μM and 35 μM), endothelial cell migration was inhibited by 50% and 70% respectively (Figure 4A and 4B). These results were further confirmed by real-time monitoring of VEGF-A induced endothelial cell (HUVEC-I) migration by electrical impedance measurements using trans-well plates. As shown in Figure 4C, iron decreased the rate (slope) of migration of endothelial cells in response to VEGF-A in a concentration-dependent manner. Reduced migration was evident at 9 hours following treatment with FAC. Iron (35 μM) completely inhibited cell migration towards a VEGF-A gradient (Figure 4C).

**Figure 5:**
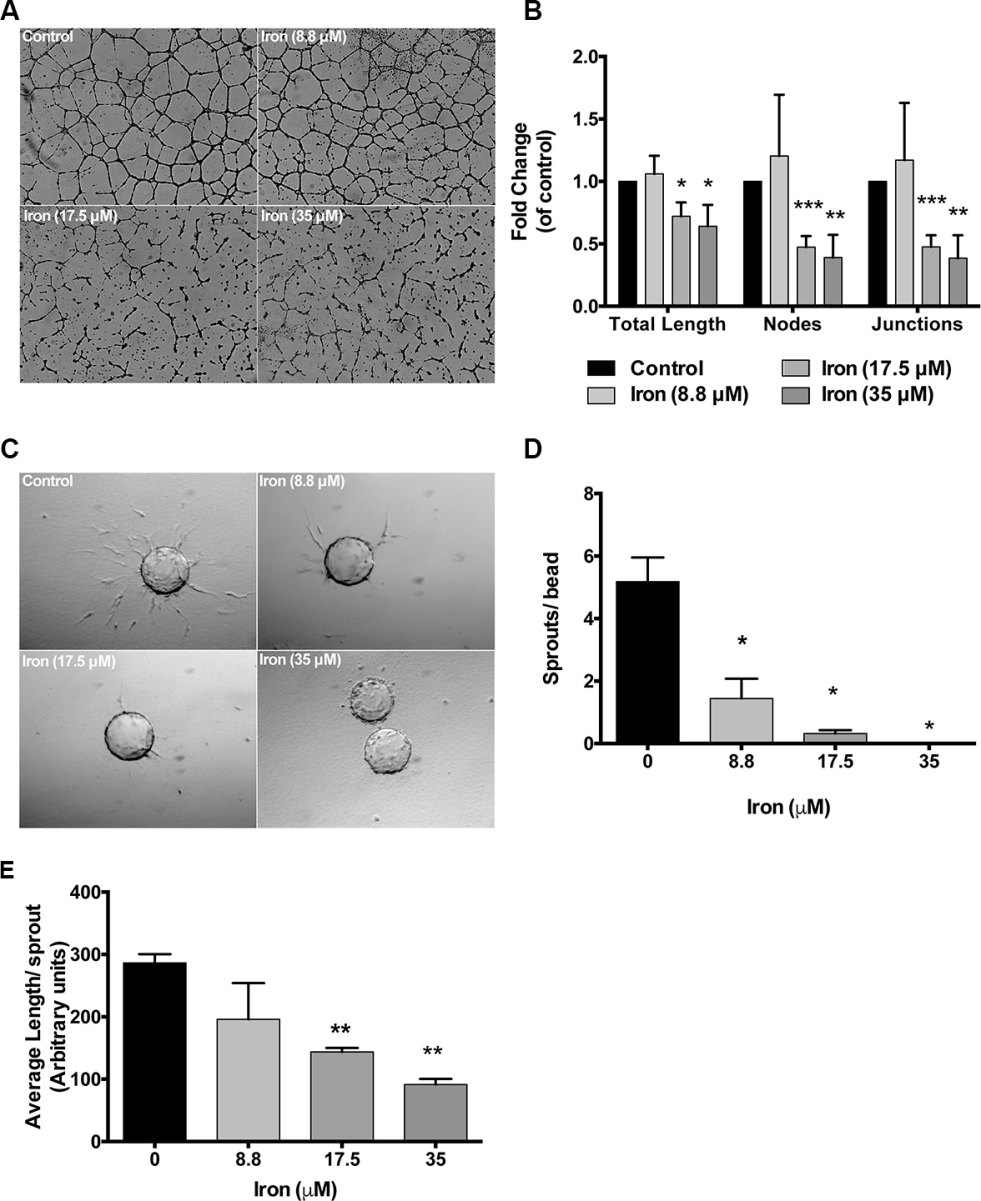
Cell-permeable iron inhibits endothelial tube formation and sprouting (**A**) Representative pictures of endothelial (HUVEC-I) tube formation in the presence of cell-permeable iron are shown (4× magnification). (**B**) The histogram represents quantification of total tube length and branch points in the presence of cell-permeable iron. Data represent mean ± SD from three independent experiments. *P < 0.05, **P < 0.01, ***P < 0.001. (**C**) Representative images of endothelial cell sprouts (HUVEC) in a growth factor-rich fibrin matrix in the presence of cell-permeable iron (10× magnification) are shown. (**D** and **E**) The histograms represent mean number of sprouts (sprouts with length ≥ bead diameter) and average length of sprout (all sprouts) per bead (total of 20 beads) in the presence of cell-permeable iron. Data represent mean ± SD of two independent experiments. *P < 0.05, **P < 0.01.

Endothelial cell morphogenesis is an integral part of angiogenesis. Tube-forming assays truly reflect the complex processes of cell migration and rearrangement to establish a network of cells with a lumen. Effect of FAC was then studied on tube formation. There was a concentration dependent effect of iron on endothelial tube formation. While low concentrations of cell-permeable iron (8.8 μM) did not inhibit, higher concentrations (17.5 μM and 35 μM) significantly inhibited endothelial tube formation. In addition to a decrease in the total tube length, there was a reduction in the number of branch points (nodes and junctions) (Figure 5A and 5B). These results were further confirmed in three-dimensional endothelial cell-sprouting assays. Cytodex™3 micro carriers beads were coated with endothelial cells and then embedded in a growth factor rich fibrin matrix. Iron treatment inhibited sprouting of endothelial cells (primary HUVEC) in a concentration-dependent manner. Low concentrations of cell-permeable iron (8.8 μM) decreased the number of sprouts per bead but did not affect the sprout length. Higher concentrations of cell-permeable iron (17.5 μM and 35 μM) significantly decreased the number of sprouts and the sprout length (Figure 5C–5E). Endothelial cells in the presence of 35 μM iron failed to produce any viable sprouts (Figure 5D).

### Cell-permeable iron inhibits VEGFR-2 phosphorylation and decreases VEGF-A signaling

**Figure 6:**
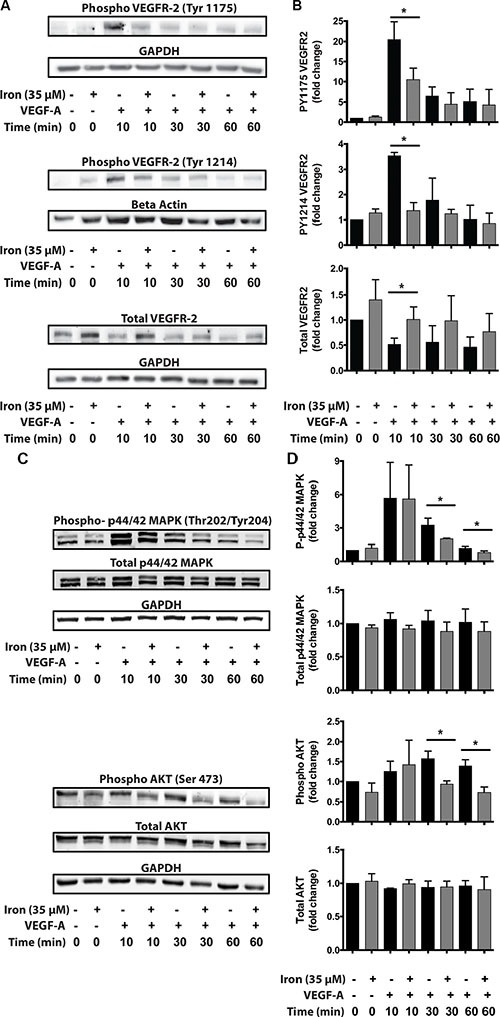
Cell-permeable iron inhibits VEGFR-2 receptor phosphorylation and downstream signaling HUVEC-I cells were stimulated with VEGF-A (100 ng/ml) for 10 min to 60 min in the presence of 35 μM iron. (**A**) Cell lysates were probed for phosphorylated tyrosine residues (pTyr-1175 and pTyr-1214) by Western blots using site-specific antibodies. Representative blot from two-three independent experiments is shown. (**B**) Densitometry analyses were used to determine relative levels of phosphorylated VEGFR-2 and total VEGFR-2. GAPDH levels were used for normalization for pTyr-1175 and total receptor levels. Beta-Actin levels were used for normalizing pTyr-1214 VEGFR-2 levels. Data represent mean ± SD from two-three independent experiments. (**C**) Representative Western blot showing phosphorylated p44/p42 MAPK and phosphorylated AKT. (**D**) Densitometric analysis of relative levels of phosphorylated and total p44/p42 MAPK and p-AKT. GAPDH levels were used for normalization. Data represent mean ± SD from two-three independent experiments. *P < 0.05.

In order to study the mechanism underlying inhibitory effect of iron on VEGF-A induced proliferation and migration, we studied the effect of cell-permeable iron on VEGF-A induced signaling in endothelial cells (HUVEC-I). Cell-permeable iron did not affect the level of constitutive phospho-VEGFR-2 levels. When stimulated by VEGF-A, the phosphorylation of the receptor increased approximately 20-fold (Tyr-1175) and 3.5-fold (Tyr-1214) at 10 minutes. Cell-permeable iron significantly decreased this phosphorylation of VEGFR-2 at Tyr-1175 and Tyr-1214 sites to less than 50% of the control. Cell-permeable iron prevented phosphorylation of the receptor even after 30 minutes of treatment with VEGF-A. Interestingly, total VEGFR-2 levels increased with cell-permeable iron (Figure 6A and 6B). These results were further validated in the primary HUVEC (Supplementary Figure S1).

As a consequence of decreased phosphorylation at the receptor level, down stream signaling was affected by iron treatment. Mitogen Associated Protein Kinase (MAPK, p44/42) is involved in VEGF-induced proliferation and activation of AKT-pathway increases survival signals in endothelial cells. FAC did not change the constitutive levels of phospho-p44/42 MAPK or phospho AKT. When stimulated by VEGF-A, phosphorylation at both p44/42 MAPK and AKT increased approximately 5-fold and 1.5-fold respectively. FAC, however, caused a significant reduction in VEGF-induced phosphorylation of p44/42 Mitogen-Activated Protein Kinase (MAPK) and the serine/threonine specific protein kinase AKT at 30 minutes and 60 minutes. Cell-permeable iron did not alter the total levels of p44/42 MAPK and AKT (Figure 6C and 6D). These results were further validated in the primary HUVEC (Supplementary Figure S1).

**Figure 7:**
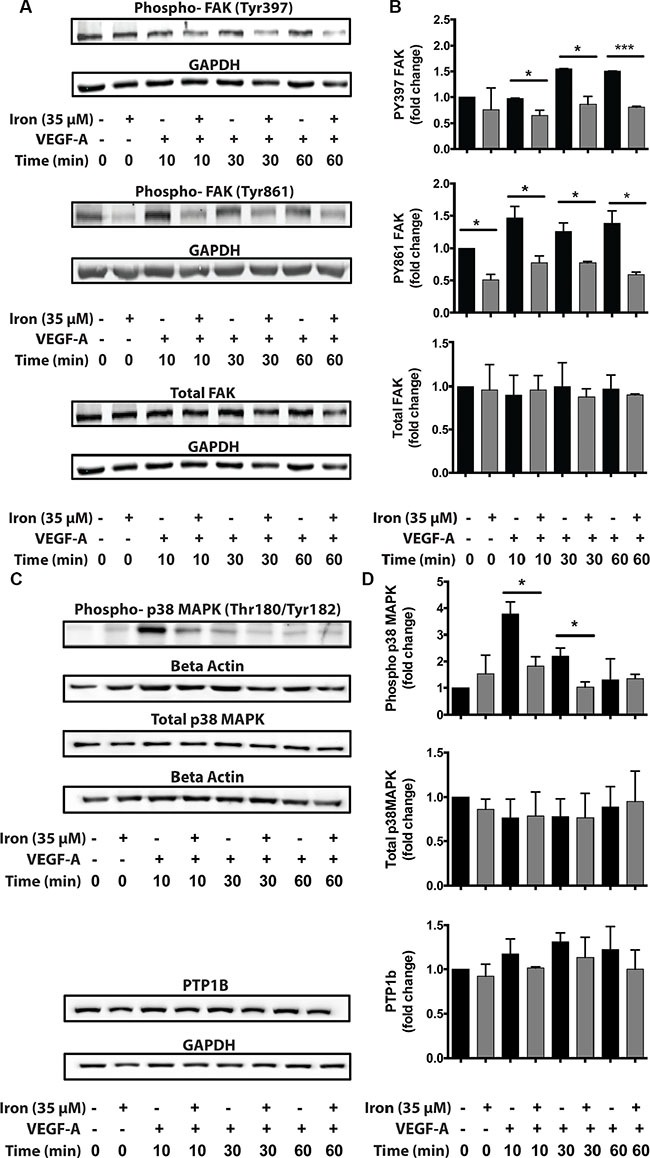
Cell-permeable iron inhibits VEGFR-2 signaling through FAK and p38 MAPK affecting migration HUVEC-I cells were stimulated with VEGF-A (100 ng/ml) for 10 min to 60 min in the presence of 35 μM iron. (**A**) Cell lysates were then analyzed by Western blots to determine relative levels of phosphorylated FAK (pTyr-397 and pTyr-861). (**B**) Cumulative data showing relative levels of FAK phosphorylation and total FAK levels from two-four independent experiments. (**C**) Western blots showing phosphorylated p38-MAPK and total levels of PTP1B following VEGF-A stimulation of HUVEC-I cells in the presence of cell-permeable iron. (**D**) Densitometric analyses of Western blots from two independent experiments. Beta-Actin levels were used for normalization of phospho and total p38 MAPK levels. GAPDH levels were used to normalize PTP1B levels. *P < 0.05, ****P < 0.0001.

Since cell permeable iron not only inhibited VEGF-induced cell proliferation but also migration, we next investigated the status of focal adhesion kinase (FAK), which is a hub of cell migration related signaling machinery. While FAC did not affect constitutive PY397 FAK levels, it decreased constitutive PY861 FAK levels. When stimulated by VEGF-A, phosphorylation increased approximately 1.5 times at both these sites (Figure 7B). Iron treatment decreased VEGF-A induced phosphorylation of endothelial Focal Adhesion Kinase (FAK) at Tyr-397 and Tyr-861 sites. Inhibition of FAK phosphorylation was evident as early as 10 minutes following treatment and sustained over a period of 60 minutes. The total levels of FAK were not changed by cell-permeable iron (Figure 7A and 7B). These results were further validated in the primary HUVEC (Supplementary Figure S1). In addition, cell-permeable iron decreased phosphorylation of p38 MAPK, providing another mechanism to hinder endothelial migration induced by VEGF-A (Figure 7C and 7D). Decreased phosphorylation of FAK could either be due to inhibition of Src-mediated phosphorylation or due to increased dephosphorylation. Protein Tyrosine Phosphatase 1B (PTP1B) has been implicated in VEGFR-2 dephosphorylation. Therefore, we investigated whether cell permeable iron increases the levels of PTP1B. Data in Figure 7C and 7D show that PTP1B levels did not change over a period of 60 minutes following VEGF-A treatment in the presence or absence of cell-permeable iron. These results suggest that dephosphorylation by PTP1B may not be involved in FAC-mediated decrease in FAK phosphorylation.

### Cell-permeable iron inhibits VEGF-induced angiogenesis *in vivo*

**Figure 8:**
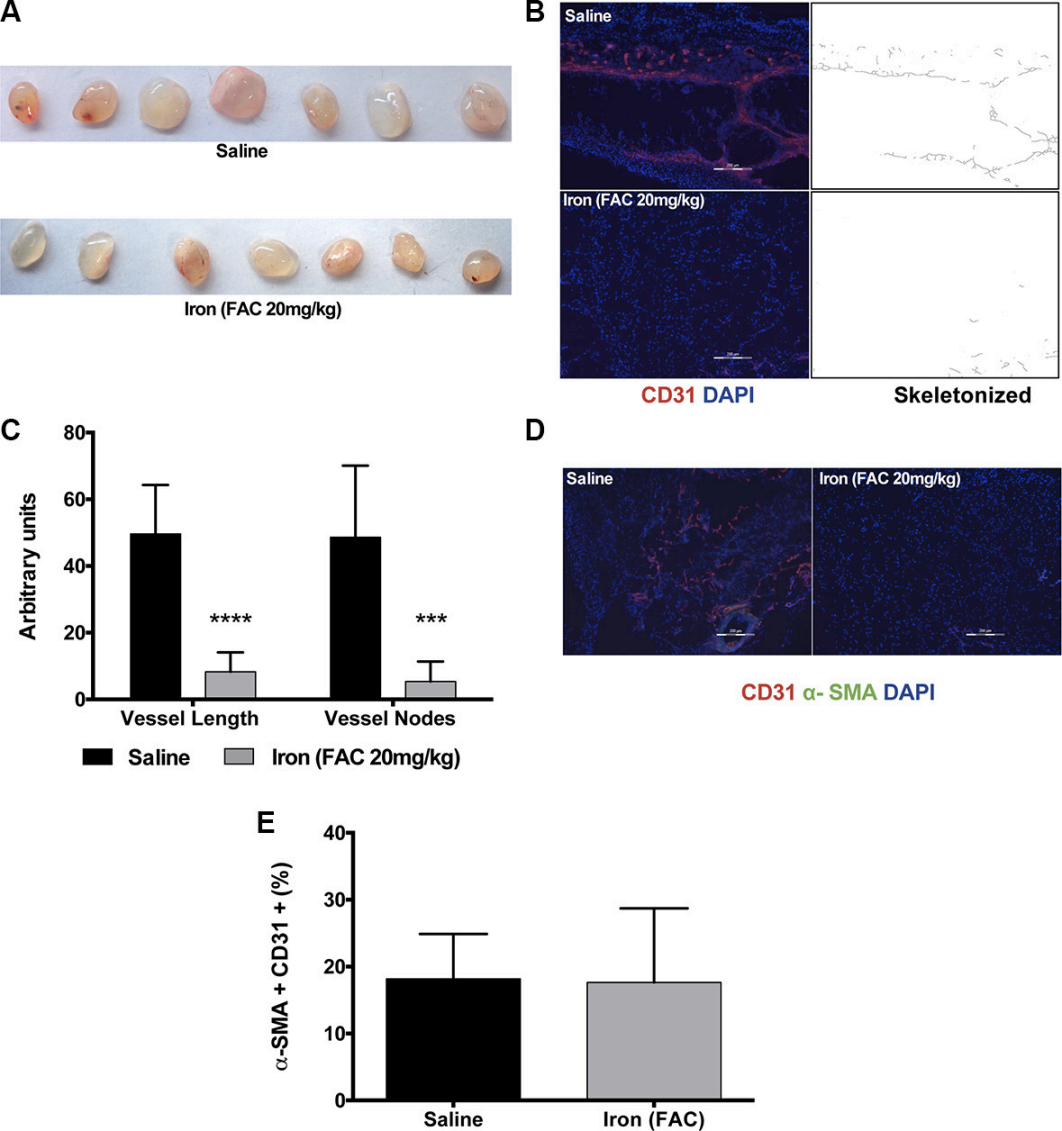
Iron inhibits VEGF-A induced angiogenesis *in vivo* Geltrex™ plugs containing VEGF-A (200 ng/ml) were used to study angiogenesis *in vivo*. (**A**) Gross morphology of the gel plugs surgically resected from mice. (**B**) Representative images of CD-31 positive vessels and corresponding binearized skeletonized images of Geltrex plugs obtained from control mice receiving saline and mice systemically treated with cell-permeable iron are shown (10× magnification). (**C**) The histogram represents average CD31-positive blood vessel length and branch points in plug sections (*n* = 7). ***P < 0.001, ****P < 0.0001. (**D**) Representative images of VEGF-A induced mature vessels formed in mice receiving saline or iron are shown (10× magnification). (**E**) The histogram represents percent of pericyte-coated mature blood vessels (α-SMA positive vessels co-staining with CD31) in plugs harvested from mice receiving saline or iron (*n* = 7).

After establishing that cell-permeable iron inhibits VEGF-A-induced signaling in endothelial cells, effects of iron on angiogenesis were determined *in vivo*. Geltrex™ plugs containing VEGF-A were implanted subcutaneously in immunocompetent mice. Vascularization of Geltrex plugs was determined after fourteen days. Figure 8A shows gross morphology of Geltrex plugs after surgical removal. Frozen sections of the plugs were then stained with an antibody to mouse CD-31 conjugated to Phycoerythrin. Immunofluorescence studies revealed decreased recruitment of CD31 positive vessels in the plugs harvested from mice treated with systemic iron therapy (Figure 8B and 8C). Pericyte coating identified mature blood vessels. Systemic iron treatment did not change α-SMA positive pericyte-coated vessels (Figure 8D and 8E).

### Cell-permeable iron inhibits tumor cell-induced angiogenesis

**Figure 9:**
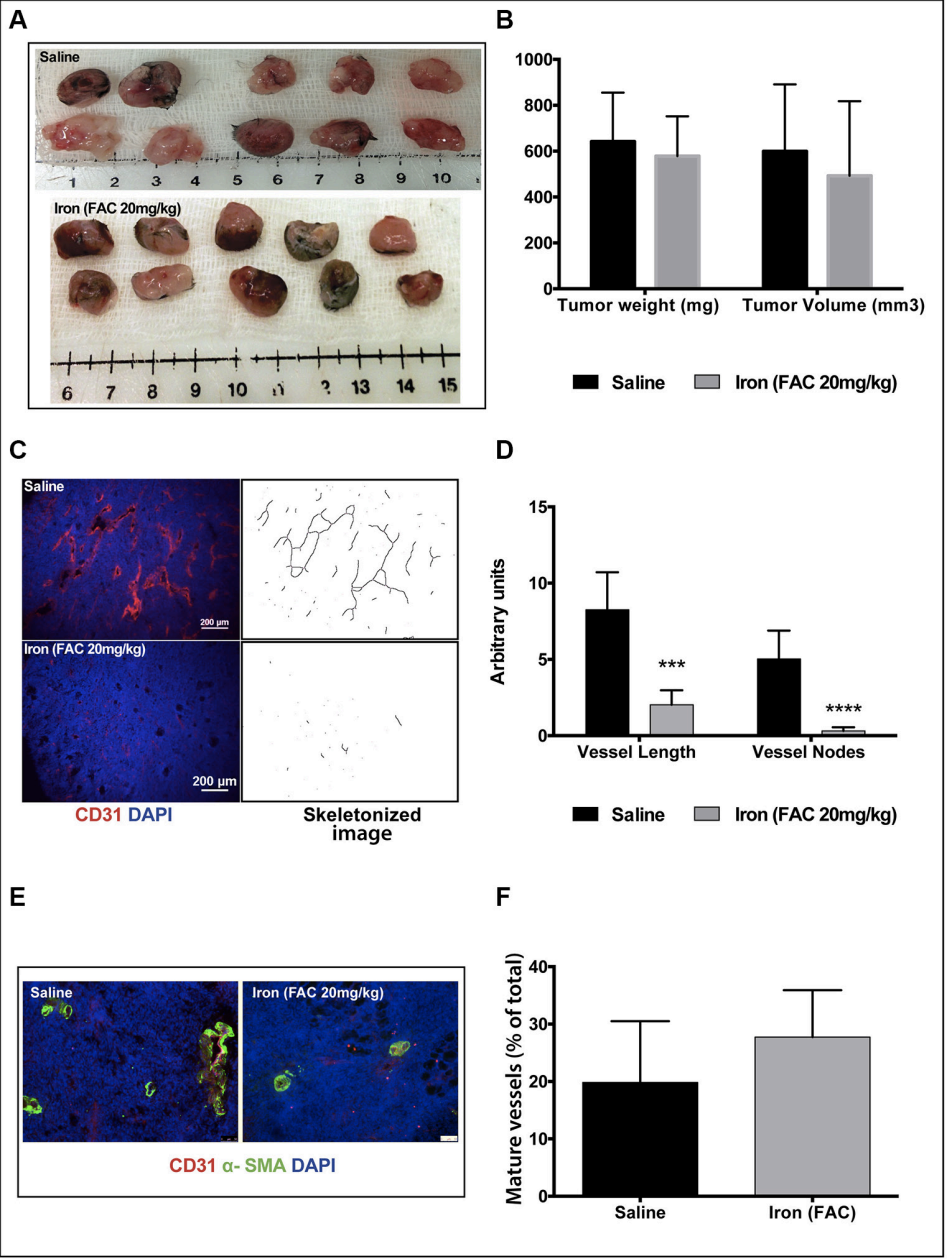
Iron inhibits tumor cell-induced angiogenesis Matrigel plugs containing syngeneic tumor cells were used for *in vivo* angiogenesis assay. (**A**) Gross morphology of the tumor plugs are shown. (**B**) The histogram shows average weight and volume of LLC tumor-matrigel plugs harvested from mice receiving saline or iron. (**C**) Representative images of CD31-positive blood vessels and corresponding binearized skeletonized images of LLC tumor-matrigel plugs are shown (4× magnification). (**D**) The histogram represents average CD31-positive vessel length and branch points in tumor matrigel plug sections (*n* = 5–7). ***P < 0.001, ****P < 0.0001. (**E**) Representative images of tumor cell-induced mature blood vessels in matrigel plugs obtained from mice receiving saline or iron are shown (20× magnification). (**F**) The histogram represents percent of pericyte-coated mature blood vessels (α-SMA positive vessels co-staining with CD31) in tumor plugs harvested from mice receiving saline or iron (*n* = 5–7).

Next, we evaluated the effect of iron therapy on tumor cell-induced recruitment of neo-vasculature in an immunocompetent mouse model using syngeneic tumor cells. Lewis Lung Carcinoma (LLC) cells were reconstituted in Matrigel plugs and subcutaneously implanted into C57/Bl6 mice. Data in Figure 9A show the gross morphology of the tumor plugs harvested from the mice after fourteen days. Figure 9B summarizes the weight and volume of the plugs. While short-term iron therapy did not affect either the volume or weight of tumor plugs, immunofluorescence staining (CD31) showed that recruitment of new blood vessels into the plugs was significantly reduced. Representative images of CD31-positive vessels from control and iron treated tumor plugs are shown in Figure 9C. Vessel length and branch points are significantly reduced by iron treatment (Figure 9D). Similar to VEGF-induced angiogenesis inhibition of Geltrex™ plugs, there was no significant difference in pericyte-coated mature blood vessels in the Matrigel tumor plugs obtained from either control or iron treated mice (20% in saline and 27% in iron treated group) (Figure 9E and 9F). These studies suggest that cell-permeable iron, which is clinically used to reduce phosphate levels in dialysis patients, can be repurposed to inhibit VEGFR-2-mediated signaling in endothelial cells and inhibit tumor angiogenesis *in vivo*.

## DISCUSSION

Iron and oxygen regulation in a cell are intertwined at many levels [11]. Iron is a cofactor for PHD, which determines the stability of HIF-1α and HIF-2α under normal oxygen levels. This notion is supported by the fact that chelation of iron by DFX stabilizes HIF under normal oxygen conditions [5, 7]. Our previous studies have shown that HIF inhibits endocytosis and iron uptake in cancer cells by inhibiting Dynamin-2, a GTPase involved in vesicle budding. This further stabilizes HIF-1α and HIF-2α. In these studies, cell-permeable iron was able to counteract the effect of Dynamin-2 inhibition on HIF-1α in tumor cells [6]. Based on these observations, we hypothesized that cell-permeable iron would perturb this regulation in hypoxic endothelium and inhibit HIF. Interestingly, cell-permeable iron did not decrease the levels of HIF-1α or HIF-2α in hypoxic endothelial cells (Supplementary Figure S2). There appears to be a cell-lineage specific difference in the effect of iron on HIF levels. This may be because of other cofactors involved in the regulation of PHD activity, namely, ascorbic acid and alpha-ketoglutarate (an intermediate in tricarboxylic acid cycle) and their differential regulation in tumor and endothelial cells. In our studies, cell-permeable iron inhibited the autocrine stimulation pathway down stream of HIF. HIF-driven VEGF-VEGFR autocrine stimulation of endothelial cells is well known. Hence, we investigated the effect of iron on VEGF receptor signaling mechanisms.

Cell-permeable iron inhibited tyrosine phosphorylation of VEGFR-2 at two critical sites-Tyr-1214 and Tyr-1175. While the phosphorylation of Tyr-1214 recruits NCK/Fyn/PAK-2 complex and activates endothelial cell migration, phosphorylation of Tyr-1175 is important for both endothelial proliferation and migration [12]. Phospho-Tyr-1175 activates Src kinase leading to phosphorylation of FAK and cell migration. The same site also provides a hub for recruiting signaling complex involved in the activation of PKC and PI3K leading to cell proliferation and survival respectively. Since, phosphorylation status of tyrosine kinase receptor is regulated by phosphatases, we determined the levels of the phosphatase PTP1B, which dephosphorylates PhosphoTyr-1175 site [13]. Our studies show that iron treatment did not alter the levels of PTP1B. Although the levels of the phosphatase did not change, it is still possible that the activity of phosphatases can be modulated by cell-permeable iron, which can then affect the status of VEGFR-2 phosphorylation. Alternatively, cell-permeable iron might interfere with VEGFR-2 internalization and receptor trafficking. Tyrosine phosphorylation of VEGFR-2 receptor at 1175 site is known to occur within the endosomal compartment [14]. Hence, inhibition of receptor trafficking would lead to decreased phosphorylation and increased dephosphorylation by PTP1B at Tyr-1175 site. Moreover, disturbance of receptor trafficking would also affect its recycling and degradation in the lysosomal compartment. This would explain why iron treatment increased levels of total VEGFR-2 in endothelial cells (Figure 6). In our studies, DFX reversed the effects of cell-permeable iron on VEGF-A induced proliferation (Figure 1E and 1F). These results complement with studies showing a stimulatory effect of DFX on angiogenesis and wound healing [15–17]. This underscores the importance of iron homeostasis in endothelium and how angiogenesis can be affected by tipping the balance in either direction.

Furthermore, systemic treatment with cell-permeable iron significantly inhibited angiogenesis *in vivo*. Both growth factor-induced and tumor cell-induced angiogenesis were inhibited by iron (Figure 9). While iron treatment reduced the number of blood vessels in Matrigel plugs containing tumor cells, the number of pericyte-coated, mature vessel number was not affected by iron treatment. Maintaining mature vasculature (vessel normalization) would have an impact in chemotherapeutic drug delivery. Thus, iron treatment will not only inhibit tumor angiogenesis but may also help in vessel normalization to improve drug delivery. Most tumors develop chemo-resistance over time because of export pumps or failure of therapy to reach the stem cell niche within the tumor [18, 19]. Cell-permeable iron enters cells independent of receptor-mediated uptake mechanisms and is not subject to export pumps and hence would not be susceptible to these resistance mechanisms. Ferric citrate (similar to ferric ammonium citrate) is currently used in dialysis patients to reduce dietary phosphate absorption. Clinical studies have shown that serum levels of ferric citrate reach 15 μM with oral dosing and have been well tolerated in these patients [20]. In the present studies, ferric ammonium citrate was found to have an anti-angiogenic role at comparable concentrations. Our studies demonstrate the potential use of cell-permeable iron in inhibiting tumor angiogenesis without toxicity. Further development of cell-permeable iron however warrants detailed toxicity studies. Intraperitoneal injection of cell-permeable iron is particularly suitable for inhibiting angiogenesis in peritoneal cancers such as ovarian cancer.

**Figure 10:**
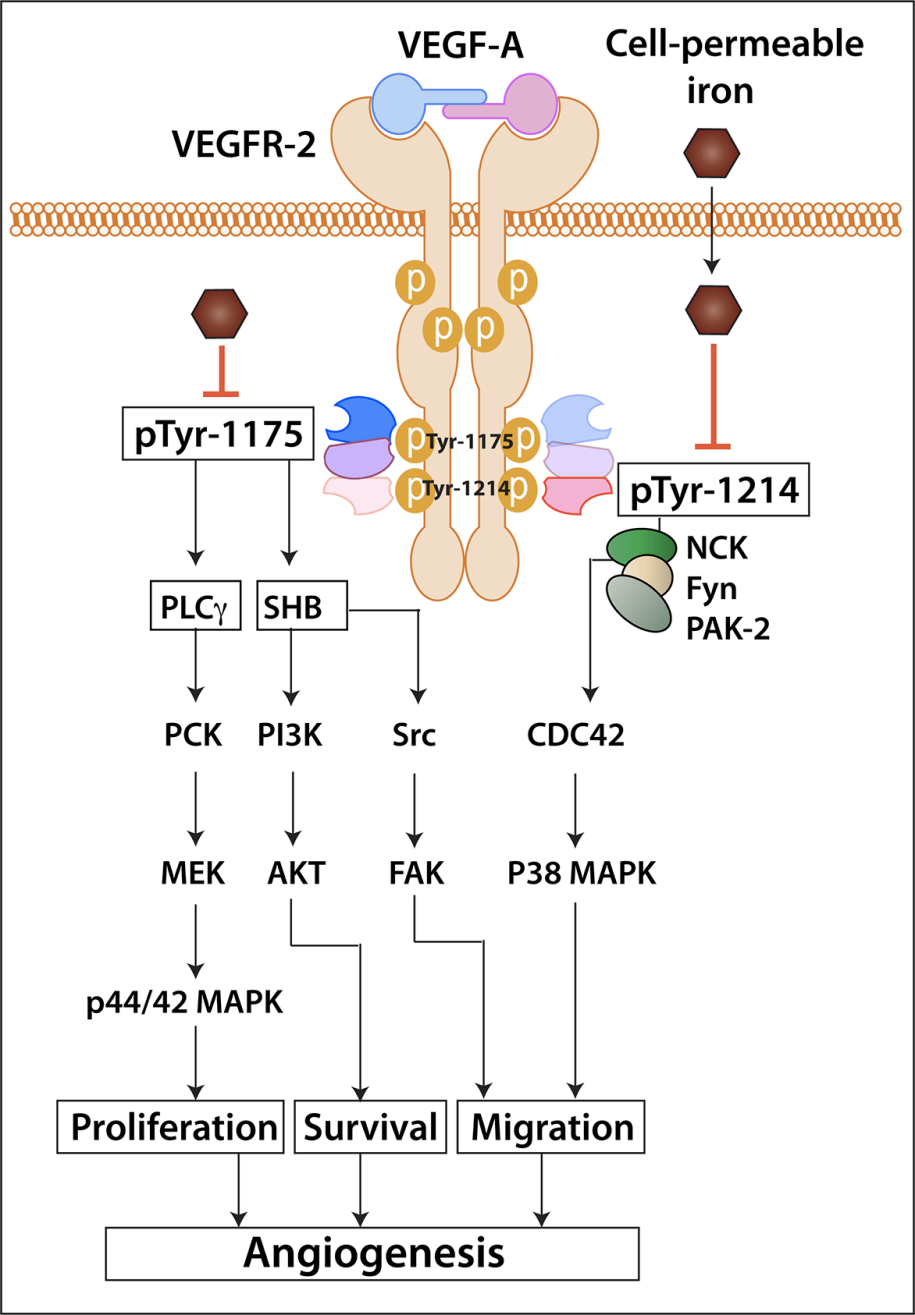
Schematic diagram shows the working model of iron-mediated inhibition of VEGFR-2 signaling pathway and angiogenesis

In summary, cell-permeable iron inhibits VEGFR-2 receptor phosphorylation and signaling in the endothelial cells. Consequently, endothelial cell proliferation, migration and viability are affected. Systemic treatment of mice with cell-permeable iron inhibits tumor angiogenesis. Schematic diagram in Figure 10 shows a working model based on these results. Additional preclinical studies are needed to evaluate whether cell-permeable iron can be used to potentiate the effects of other anti-angiogenic therapeutics.

## MATERIALS AND METHODS

### Cell culture

HUVEC-I and MVEC were provided by Dr. A. Kelekar and Dr. J. Hall respectively (University of Minnesota). Primary HUVEC cultures were purchased from Neuromics (Edina, MN). Endothelial cells were cultured in Endothelial cell Growth Media EGM-2™ (Lonza). For growth factor starvation, Endothelial cell Basal Medium-2™ (Lonza) was used. Experiments were performed on primary HUVEC obtained from two different batches and MVEC used in real-time experiments were procured from a single batch. LLC cells were obtained from ATCC (Manassas, VA) and grown in RPMI 1640 medium supplemented with 10% FBS, 1% glutamine, 1% Penicillin-Streptomycin and 1% sodium pyruvate.

### Treatment with FAC

Endothelial cells were treated with cell-permeable iron (FAC). FAC has an iron content of 17.5% (16.5–18.5%) and hence iron concentration for experiments was normalized accordingly.

### Reagents

VEGF-A (Isoform 165) was cloned and expressed in yeast [21]. Geltrex^®^ Low-growth factor basement membrane matrix (Life Technologies) was used for *in vitro* tube formation assays. Matrigel™ basement membrane matrix (BD Biosciences) was used for tumor cell-induced angiogenesis studies. Cytodex 3™ micro carrier beads (GE Healthcare Life Sciences) were used for fibrin bead assay. Antibodies used were: anti p44/42 MAPK (CST#4696S), anti phospho-p44/42 MAPK (CST#4370S), anti VEGFR-2 (FLK-1) (sc-2651), anti phospho-Tyr 1175 VEGFR-2 (CST#2478S), anti phospho-Tyr 1214 VEGFR2 (ab5475), anti AKT (CST#2920S), anti phospho-Ser 473 AKT (CST#3787S), anti FAK (EMD Millipore 05-537), anti phospho-Tyr 397 FAK (CST#3283S), anti phospho-Tyr 861 FAK (ab4804), anti p38 MAPK (CST#9211S), anti phospho-p38 MAPK (CST#4511S), anti GAPDH (MAB374), anti Beta-Actin (sc-47778), anti PTP1B (sc-1718), anti HIF-2α (sc-13596), anti HIF-1α (NB 100-479), PE conjugated anti-CD31 (BD Pharminogen-52477), FITC-conjugated anti-αSMA (Sigma-F377), FITC-conjugated anti-cleaved PARP (Asp 214, BD Biosciences) and FITC-conjugated anti-cleaved caspase-3 (Asp 175, BD Biosciences). Appropriate secondary antibodies conjugated to horseradish peroxidase (Vector labs) or IRDye^®^ secondary antibodies (LI-COR) were used. Fluorescent probes-Hoescht-33342, 4′, 6-diamidino-2-phenylindole (DAPI) and PI were purchased from Molecular Probes (Invitrogen). FAC and DFX were obtained from Sigma. Cell Counting Kit-8 (CCK-8) was purchased from Dojindo Molecular Technologies.

### VEGF-A induced proliferation

HUVEC-I (8,000–10,000) were plated in gelatin (0.2%) coated 96 well plates and allowed to attach for 24 hours. Quiescent cultures were treated with different concentrations of iron in EGM-2. All treatments were done in triplicates. Cell viability was measured by using WST-8 (CCK-8) assay. Parallel series of experiments were carried out to determine the effect of cell-permeable iron on VEGF-A induced proliferation of endothelial cells. In these experiments, HUVEC-I cells were seeded and growth factor starved (EBM-2 with 2% serum) for 16 hours. Then cultures were induced to proliferate in the presence of 100 ng/ml VEGF-A in EBM-2 medium containing 2% serum. Cell viability was determined by CCK-8 assay kit. VEGF-A induced proliferation was calculated by subtracting negative control values from all experimental groups. Iron-induced inhibition of endothelial cell proliferation was calculated as a percentage of the positive control (VEGF-A).

### Real-time endothelial cell proliferation

HUVEC-I (20,000) and MVEC (20,000) were plated in gelatin (0.2%) coated E-plate 16 (ACEA Biosciences). Growth factor starvation and VEGF-A treatment were done as previously described. Two independent experiments were carried out. Impedance measurement was recorded every 15 minutes for 48–72 hours using the Real-Time Cell Analyzer Dual Plate (RTCA DP) Instrument (ACEA Biosciences).

### Cell cycle analysis

HUVEC-I cells were plated in gelatin (0.2%) coated 60 mm dishes. Once 75% confluent, they were growth factor starved in EBM-2 with 1% serum overnight to induce G_0_ cell cycle arrest. After 16 hours of starvation, cells were stimulated to proliferate in EGM-2 complete medium in the presence of 17.5 μM and 35-μM iron for 24 hours and 48 hours. Cells were then fixed in ice-cold 70% ethanol and stained with PI (20 μg/ml) and RNase (200 μg/ml) for 30 min at room temperature. Cells labeled with PI were then analyzed in BD FACS Canto II using FACS Diva software (BD Biosciences, USA) and 10,000 events were recorded.

### Cell death analysis

HUVEC-I were growth factor starved and treated with different concentrations of iron in EBM-2 supplemented with 100 ng/ml VEGF-A for 24 and 48 hours, as previously described. At the end of experiment, cells were stained with Hoescht-33342 (12 μg/ml) and PI (2.5 μM) and images were captured at 10× magnification using Leica DMI3000B. Using ImageJ software, cell death was quantified as percentage of PI positive nuclei out of the total Hoescht-33342 positive nuclei.

### Flow cytometric detection of cleaved caspase-3 and cleaved PARP

HUVEC-I cells were plated in gelatin (0.2%) coated 60 mm dishes. They were growth factor starved and treated with different concentrations of iron in EBM-2 supplemented with 100 ng/ml VEGF-A for 48 hours, as previously described. Doxorubicin (4 μM) was used as a positive control for induction of apoptotic cell death. Cells were harvested, fixed and stained with FITC-conjugated monoclonal antibodies for cleaved PARP and cleaved caspase-3, as previously described [22]. Cells were analyzed in BD FACS Canto II using FACS Diva software (BD Biosciences, USA) and 5,000 events were recorded.

### VEGF-A induced cell migration (scratch wound assay)

HUVEC-I (50,000) were plated in gelatin-coated (0.2%) 24 well plates and allowed to attach for 24 hours. Subsequently they were growth factor starved in EBM-2 with 2% serum overnight and a scratch wound assay was carried out, as previously described [23]. The cells were treated with 17.5 μM and 35-μM iron in EBM-2 with 2% serum and 100 ng/ml VEGF-A. Images were captured at time zero and at 24-hour after preparing scratch wounds at 2.5× and 4× magnification using a Nikon AZ100M Fluorescence Microscope. Closure of the wound area was quantitated by ImageJ software.

### VEGF-A induced migration (real-time trans-well migration)

HUVEC-I cells (50,000) were plated in the upper chamber of the CIM Plate-16 (ACEA Biosciences) in the presence of 17.5 μM and 35-μM iron in serum free medium. Migration of cells was monitored in real-time towards a gradient of 100 ng/ml VEGF-A (lower chamber). Impedance measurement was recorded every 15 minutes for 24 hours using the Real-Time Cell Analyzer Dual Plate (RTCA DP) Instrument (ACEA Biosciences).

### Tube formation assay

HUVEC-I were plated in gelatin (0.2%) coated 60 mm dishes and pre-treated with different concentrations of iron for 24 hours. Geltrex^®^ Low-growth factor basement membrane matrix (250 ul/well) was added to the wells of a 24 well plate. Subsequently, 75,000 live HUVECs were added over the matrix layer and treated with different concentrations of iron in EGM-2 for 16 hours. At 16 hours, images were captured at 4× magnification with the Nikon AZ100M Microscope and the tube length, nodes and junctions were quantitated using the ImageJ Angiogenesis Analyzer plugin.

### Fibrin bead assay

Primary HUVEC (between passages 2 and 4) were maintained in EGM-2 media and seeded on Cytodex™3 micro carriers beads for endothelial cell sprout formation as previously described with some modifications [24]. Vessel sprouting was maintained by addition of EGM-2 media with and without iron (8.8 μM, 17.5 μM and 35-μM) alternate day for a period of 5 days. Sprouts were imaged on day 5 at 10× magnification using Leica DM IL microscope. Sprout number and sprout length were quantified using ImageJ. Only sprouts with lengths greater than bead diameter were included for quantification of sprout number.

### VEGF-A mediated signaling

Endothelial cells (HUVEC, HUVEC-I) were plated in gelatin (0.2%) coated 60 mm dishes. Once 75% confluent, they were growth factor starved in EBM-2 with 2% serum overnight in the absence or presence of 35-μM iron. After 16 hours of starvation, they were stimulated with 100 ng/ml VEGF-A and lysates were collected at 0, 10, 30 and 60 min. Cells were lysed with 100 μL RIPA buffer (Alfa Aesar) containing protease and phosphatase inhibitor cocktail (Thermo Scientific). Protein concentration was measured using BCA™ Protein Assay Kit (Pierce) and 30 μg of protein was loaded. The samples were resolved in SDS-PAGE gels (7.5% and 15%) and transferred to a nitrocellulose membrane (BIO-RAD). Blots were blocked with 5% non-fat milk or 5% BSA in TBST (for phospho-proteins) for one hour at room temperature. Membranes were incubated with primary antibodies in their recommended dilutions overnight at 4°C. Membranes were then incubated with respective anti-mouse, goat or rabbit IgG secondary antibodies (1:3000) conjugated with horseradish peroxidase or anti-rabbit 680 and anti-mouse 800 IRDye^®^ secondary antibody (1:10,000) at room temperature for one hour. Immunoblots were detected with Clarity™ Western ECL Substrate (BIO-RAD) using enhanced chemiluminescence technique and IR fluorescence was detected using Odyssey Imager. Densitometric analyses and quantification of bands were done using ImageJ software.

### Geltrex™ plug assay

Low-growth factor basement membrane matrix Geltrex™ with added VEGF-A (200 ng/ml) was injected subcutaneously into the dorsal flanks of 6–8 week old female C57BL/6 wild-type mice. The University of Minnesota animal care and use committee approved all animal studies. Geltrex™ was reconstituted with or without 35-μM iron. Next day, one group was treated with 20 mg/kg FAC (in PBS) alternate day intraperitoneally. After 2 weeks of treatment, mice were sacrificed under pressurized CO2 and Geltrex™ plugs (*n* = 7 for both saline and Iron) were surgically removed and snap-frozen in liquid nitrogen. Frozen plugs were sectioned (15 mm), fixed in cold acetone (–20°C) for 10 min, rehydrated and blocked in 1% BSA. Slides were then stained with PE-conjugated anti-mouse CD31 (1:100), FITC-conjugated anti α-SMA (1:500) and counterstained with DAPI. Random areas (5-20) were imaged at 10× magnification using Zeiss Axioplan 2 Fluorescence microscope. CD31-positive vessel length and branch points (nodes) were quantified by morphometric analyses, as previously described [25]. CD31-positive vessels co-staining with α-SMA were quantified similarly to determine relative percent of mature vessels.

### Tumor cell-induced angiogenesis

Female, 6–8 weeks old, C57BL/6 wild-type (WT) mice were used for tumor cell-induced angiogenesis studies. Lewis Lung Carcinoma (LLC) cells (1 million/ plug) were mixed with Matrigel™ (3:1) and injected subcutaneously (bilateral sites) into the dorsal flanks of 6-8 week old female C57BL/6 mice. Matrigel was reconstituted with or without 35-μM iron. When the plugs were visible in all mice (day 4), one group of five mice was treated with 20 mg/kg FAC (in PBS) by intraperitoneal injections on alternate days. Control group of five mice were treated with similar volume of sterile Hanks Balanced Salt Solution following a similar schedule. After 2 weeks of treatment, mice were sacrificed by pressurized CO2 as per approved animal protocol. Matrigel plugs were resected and snap frozen in liquid nitrogen. Frozen Matrigel plugs were then sectioned and processed for CD31 and α-SMA staining, as previously described.

Random areas (5-20) were imaged at 4× and 20× magnification using Nikon AZ100M Fluorescence Microscope and Leica DM5500 B microscope. CD31-positive vessel length and branch points (nodes) were quantified by morphometric analyses, as previously described [25]. CD31-positive vessels co-staining with α-SMA were used to determine relative abundance of mature blood vessels.

### Statistics

Data are expressed as means ± SD. Data represent an average of two-four independent experiments. Statistical analyses were performed using GraphPad Prism^®^ 6. Differences in mean values between the two groups were analyzed using the two-tailed Student's *t*-test. *P* value < 0.05 was considered statistically significant.

## SUPPLEMENTARY MATERIALS FIGURES


