# Supplementary material for: Cell-permeable iron inhibits vascular endothelial growth factor receptor-2 signaling and tumor angiogenesis
Source: Oncotarget. 2016 Aug 30;7(40):65348–63. doi: 10.18632/oncotarget.11689 (PMC5323160; doi:10.18632/oncotarget.11689)
Supplement: Supplementary file 1 [file oncotarget-07-65348-s001.pdf]

Cell-permeable iron inhibits vascular endothelial growth factor receptor-2 signaling and tumor angiogenesis

Supplementary Materials

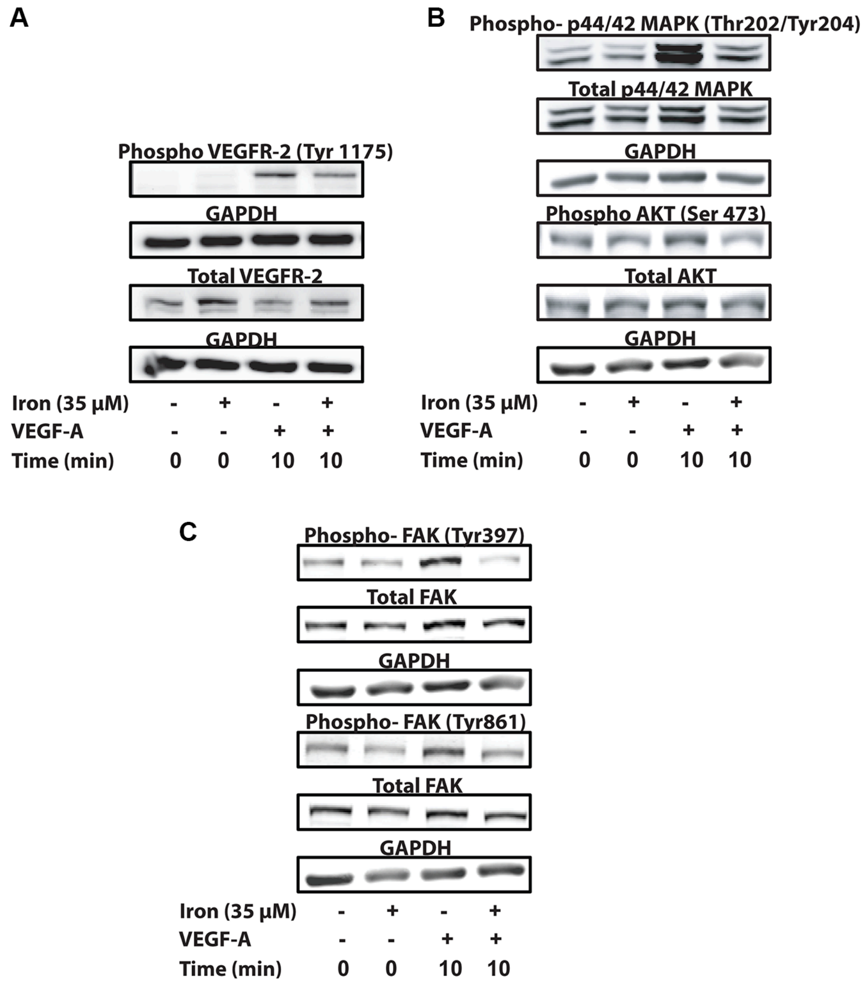

Supplementary Figure S1: Representative immunoblots of HUVEC stimulated with VEGF-A (100 ng/ml) for 10 min in the presence of 35 µM iron are shown.

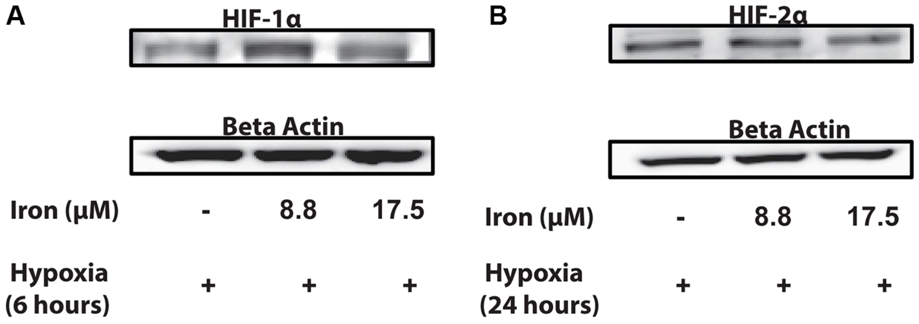

Supplementary Figure S2: Representative HIF-1α and HIF-2α immunoblots of HUVEC exposed to hypoxia in the presence of 8.8 µM and 17.5 µM iron for 6 and 24 hours respectively are shown.
